# Secure federated learning for Alzheimer's disease detection

**DOI:** 10.3389/fnagi.2024.1324032

**Published:** 2024-03-07

**Authors:** Angela Mitrovska, Pooyan Safari, Kerstin Ritter, Behnam Shariati, Johannes Karl Fischer

**Affiliations:** ^1^Fraunhofer-Institut fur Nachrichtentechnik, Heinrich-Hertz-Institute (HHI), Berlin, Germany; ^2^Bernstein Center for Computational Neuroscience, Berlin, Germany; ^3^Department of Psychiatry and Psychotherapy, Charite – Universitatsmedizin Berlin (corporate member of Freie Universitat Berlin, Humboldt-Universitat zu Berlin, and Berlin Institute of Health), Berlin, Germany

**Keywords:** Federated Learning, Secure Multi-party Computation, Alzheimer's Disease, Secure Aggregation, Machine Learning, neuroimaging

## Abstract

Machine Learning (ML) is considered a promising tool to aid and accelerate diagnosis in various medical areas, including neuroimaging. However, its success is set back by the lack of large-scale public datasets. Indeed, medical institutions possess a large amount of data; however, open-sourcing is prevented by the legal requirements to protect the patient's privacy. Federated Learning (FL) is a viable alternative that can overcome this issue. This work proposes training an ML model for Alzheimer's Disease (AD) detection based on structural MRI (sMRI) data in a federated setting. We implement two aggregation algorithms, Federated Averaging (FedAvg) and Secure Aggregation (SecAgg), and compare their performance with the centralized ML model training. We simulate heterogeneous environments and explore the impact of demographical (sex, age, and diagnosis) and imbalanced data distributions. The simulated heterogeneous environments allow us to observe these statistical differences' effect on the ML models trained using FL and highlight the importance of studying such differences when training ML models for AD detection. Moreover, as part of the evaluation, we demonstrate the increased privacy guarantees of FL with SecAgg via simulated membership inference attacks.

## 1 Introduction

Artificial intelligence's (AI) success in aiding medical diagnosis and treatment depends mainly on the publicly available diverse datasets. Many publicly available biomedical datasets are small and stem from relatively few institutions, with similar distribution in terms of demographics, offering only a limited possibility to study medical conditions with respect to the global population (Kaissis et al., 2020). One of the main reasons behind the lack of large and diverse biomedical public datasets is that patients' data is considered privacy-sensitive and has rigorous requirements for protection. Several privacy regulations, such as the Health Insurance Portability and Accountability Act (HIPAA^1^) and the General Data Protection Regulation (GDPR^2^), impose strict rules regarding sharing and storing data from different institutions. Per the privacy regulations, sharing and storing data from different institutions in a central location for further data utilization is not a viable solution. Additionally, medical datasets have a significant business value, making it less likely that they would be freely shared (Rieke et al., 2020).

Secure and privacy-preserving AI offers techniques to help bridge the gap between personal data protection and data utilization for research, as well as for aiding medical diagnosis and treatment (Kaissis et al., 2021). Federated Learning (FL) (McMahan et al., 2017), a method under privacy-preserving AI, allows for collaborative data utilization without sharing or revealing the data itself. FL allows the training of Machine Learning (ML) models locally on the site where the data is stored, and only the model parameter updates are sent to a central server for aggregation. The central server merges the model parameter updates from different participants and sends the new model parameters back to the participants for the next round of training. The main benefit of FL is the ability of the data to remain with its owner while still enabling the training of ML algorithms on remote data (McMahan et al., 2017; Kaissis et al., 2020). FL is the most widely used privacy-preserving technique, both in industry (Konečný et al., 2017) and medical AI applications (Rieke et al., 2020). FL methods have been used practically by major companies (Bonawitz et al., 2019; Goetz et al., 2019; Li et al., 2020a), and in several different applications where the training data is distributed among multiple data owners, such as in IoT (Zeng et al., 2021; Zhou et al., 2021), telecommunications (Safari et al., 2020), healthcare (Liu et al., 2018; Huang et al., 2019; Li et al., 2019; Roy et al., 2019; Rieke et al., 2020; Kaissis et al., 2021), etc. FL generally has a great perspective in medical and industrial applications due to the possibility for data utilization while abiding by existing laws and regulations (such as GDPR). FL can handle data in cross-organizational architecture, as well as data split across separate institutions, allowing the data owners to keep their data locally. However, FL is a novel technique in development that still carries crucial core challenges and open questions.

The most common algorithm used to aggregate the updates of different participants in an FL round is the Federated Averaging (FedAvg) algorithm (McMahan et al., 2017). However, FL with FedAvg lacks an inherent privacy guarantee, and other methods must be utilized to guarantee privacy. Neural Networks (NNs) are also a form of memory mechanism that store compressed representations of the training data within their parameters (weights). This could make it possible to reconstruct parts of the training data (Wang et al., 2019; Kaissis et al., 2020). To avoid data leakage from the model parameters, the Secure Aggregation (SecAgg) protocol can be used. SecAgg utilizes the concept of Secure Multi-Party Computation (SMPC) (Keller et al., 2018; Zhao C. et al., 2019) to compute sums of model parameter updates from individual participants in a secure manner, where no participant reveals their update clearly (Bonawitz et al., 2017; Safari et al., 2021). In the context of FL, SecAgg enables the computation of the new model without each participant revealing their updates to any of the other participants or the central server, offering new levels of privacy and reducing the risk of information leakage (Bonawitz et al., 2016, 2017).

FL has been utilized in the medical imaging field through tasks such as X-ray pneumonia detection (Kaissis et al., 2021), whole-brain segmentation in MRI (Roy et al., 2019; Rieke et al., 2020), COVID-19 detection (Liu et al., 2020), and analysis of different neurological diseases, such as Alzheimer's disease (AD) (Stripelis et al., 2022). AD is the most common cause of dementia in the elderly (Ritter et al., 2015). Hebert et al. (2001) predicted that by the year 2050, the number of AD patients will increase three-fold. Increasing research in the pathology and pathomechanisms involved in AD is needed to develop better treatment and diagnosis tools. Developing ML-based methods for AD diagnosis is an essential step in the evolution of methods that can diagnose AD with high accuracy (Wen et al., 2020).

In this work, we demonstrate the usage of FL with SecAgg for the training of an ML model for AD detection based on structural MRI (sMRI) data from the Alzheimer's Disease Neuroimaging Initiative (ADNI^3^) dataset. The contribution of our work is three-fold. First, we propose the usage of FL with SecAgg when training a classification algorithm for AD based on sMRI data. Second, we compare the classification performance of ML models trained using FL with FedAvg, and with SecAgg when having clients with statistically different datasets. The statistical differences taken into account in our study are the dataset size and demographical characteristics, including sex, age, and diagnosis. Our simulated heterogeneous environments take advantage of the biases most commonly affecting ML models trained on neuroimaging data (Larrazabal et al., 2020). Third, through simulated membership inference attacks, we show that our FL architecture, with the extension of SecAgg provides an extensive privacy guarantee on the client level.

## 2 Materials and methods

### 2.1 Federated learning

As the amount of available data worldwide increases, the centralization of data and algorithms has become increasingly popular. In such a setup, the data owners send their data to a central location, where a second party deals with the cleaning, processing, and fusing of data. An ML model is then trained on the data in the central location and later used for the prediction on unseen data. This concept is known as Centralized Learning (CL), where different data owners can share their local data with a centralized server to enable the training of ML models on more significant amounts of data. However, this approach doesn't come without issues: the data must be shared to a central location, exposing it to possible attacks or thefts. When the computation is centralized, the dataset can be manipulated, information regarding individuals can be derived, and the training data can be stolen (AbdulRahman et al., 2020; Kaissis et al., 2020). On the other hand, the model owners also expose their possibly unique algorithms to attacks (Kurita et al., 2020). Additionally, laws such as the GDPR aim to protect users' data privacy and security and require the data owners or users to be the absolute owners of their data. Due to CL's architecture, obeying the laws imposed to secure and protect personal data is impossible. FL is offered as a solution to take a step further in preserving the privacy of the data.

FL (McMahan et al., 2017) is a method that attends to protecting the data, allows for dealing with distributed data that is not representative of the population distribution (i.e., non-IID data), and enables collaborative learning. The first study of FL (McMahan et al., 2017) introduced the FedAvg algorithm, which combines local stochastic gradient descent (SGD) on each client with a server that performs model averaging. FedAvg is the baseline of FL in many research areas (Li L. et al., 2020). A typical implementation of the FedAvg algorithm is given in the Supplementary material.

**Figure 1:**
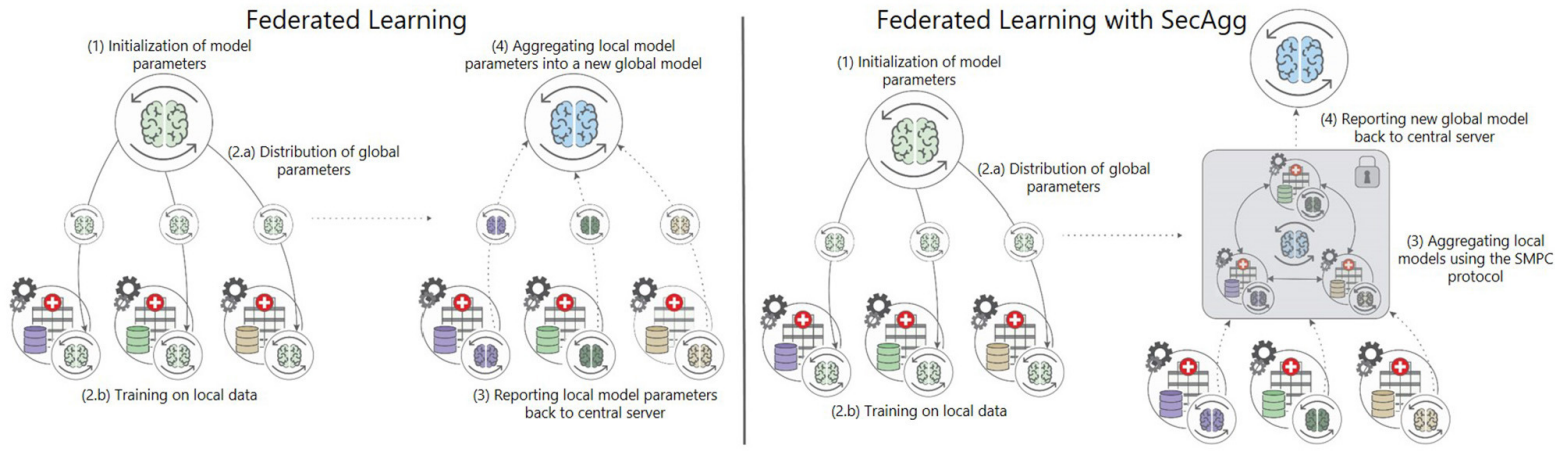
Comparison of FL with and without SecAgg. Unlike simple FL, where FedAvg is used for aggregation, in FL with SecAgg, the aggregation is done using SMPC, where all the clients collaborate to aggregate their model parameter updates.

The FL training approach is most commonly composed of several phases involving client-server communication. The process of training during an FL session is the same each epoch. It is composed iteratively of: (1) initialization of the clients, (2) distributing the global model parameters and training on local data, (3) reporting the local model parameters back to the central server, (4) aggregating and averaging the local model parameters into a new global model, and (5) sending the new global model parameters back to the clients. Essentially FL allows *N* clients to collaboratively train a global model on their local data (McMahan et al., 2017). In the process, a single client *P*_*i*_ for *i*∈*N*, doesn't share their local dataset *D*_*i*_. The global objective at round *r*>0 is to learn a model with parameters $\omega_{fed}^{r}$ from the data that is stored on the *N* clients. First, the server sends the current global model $\omega_{fed}^{r-1}$, to all participants. The clients then update the model on their local data, producing new local models $\omega_{i}^{r}$. After the local updates of the model, the models are sent back to the server, where they are aggregated into a new $\omega_{fed}^{r}$ using the FedAvg algorithm in the case of simple FL, as shown in Figure 1. In a successful federated training scenario, $\omega_{fed}^{r}$, should be reasonably close to $\omega_{cen}^{r}$, which would be the model trained using a CL approach, i.e., if all the participants shared their data in a centralized location. After the computation of the new global model, the server sends it back to the clients, and the process repeats (McMahan et al., 2017).

### 2.2 Secure Aggregation

One of the main challenges of FL with FedAvg is that it is not fully privacy-preserving by itself. If the local models are not encrypted, the data can leak, and the models can be interfered with. The input data can also be reconstructed from the NNs' parameters (weights). This is unacceptable from the data security perspective; thus, other methods must be utilized. SecAgg (Bonawitz et al., 2016) is the problem of computing a multiparty sum of the clients' model parameter updates in a federated setting without revealing the updates to the other clients or the central server. SecAgg is based on SMPC (Yao, 1982), a cryptographic primitive that enables multiple clients to jointly compute a value without revealing their private information to each other. In this study, the SecAgg implementation builds upon the idea of SPDZ (Damgård et al., 2012), one of the most popular arithmetic secret-sharing schemes under SMPC.

The SPDZ protocol is an arithmetic secret-sharing SMPC protocol in which *N* clients attempt to perform secure computation. The computation is performed over a fixed finite field, i.e., modulo prime *Q*. In SecAgg, each of the clients *P*_*i*_, after updating their local model ω_*i*_, encodes it to fixed-point precision and then splits the model into *N* random shares $\alpha_{j}^{i}$ within the finite field. The client *P*_*i*_ sends the share $\alpha_{j}^{i}$ to the *P*_*j*_ client. Thus, none of the clients possess the original value ω_*i*_; only shares without any value on their own that, when added, would produce the original value. The original value is unknown when *N*−1 elements are known (Evans et al., 2018). Once the clients receive the shares from all the other clients, they sum them up and send the corresponding result to the central server, as presented in Figure 1. The algorithm for FL with SecAgg is presented in the Supplementary material.

### 2.3 Federated learning-based Alzheimer's disease detection

**Figure 2:**
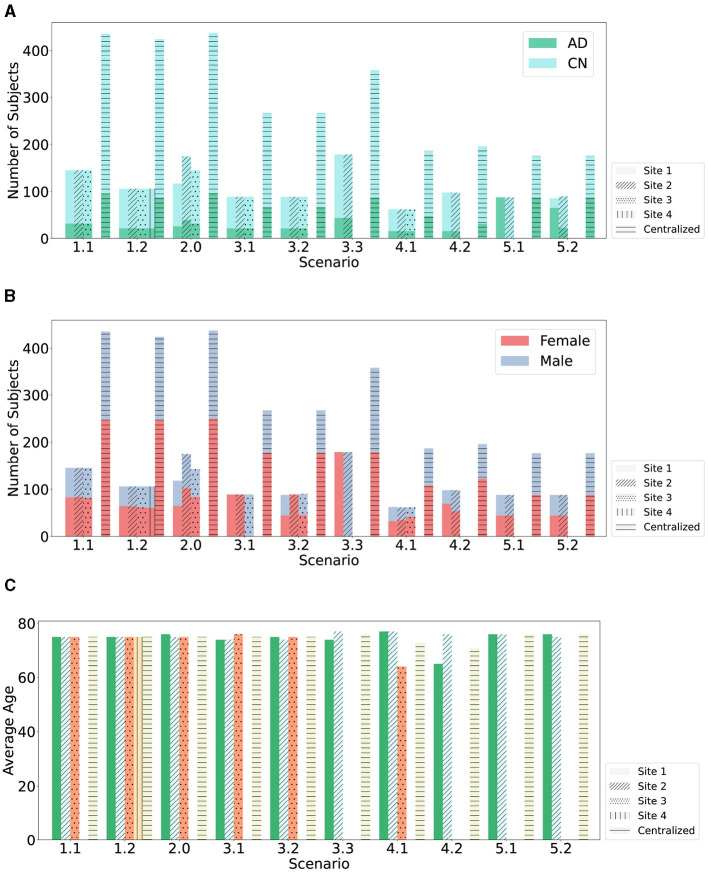
Data partitioning across clients with respect to number of subjects. **(A)** The plot shows the data partitioning across clients with respect to the two diagnosis labels (AD and CN) over the ten studied scenarios. **(B)** The plot shows the data partitioning across clients with respect to the patient's sex over the ten studied scenarios. **(C)** The plot shows the data partitioning across clients with respect to the average age over the ten studied scenarios.

**Figure 3:**
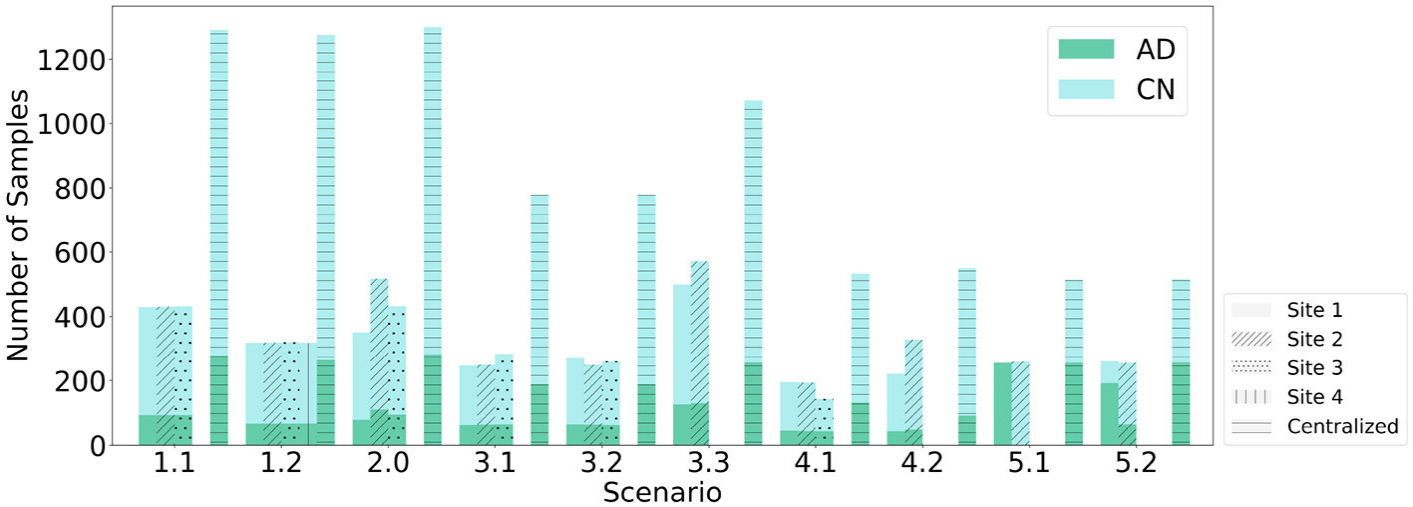
Data partitioning across clients with respect to the number of samples.

This study introduces an FL-based AD detection method based on sMRI data. We compare the Relative Performance Decrease (RPD) between models trained using CL, FL with FedAvg, and with SecAgg. Ten scenarios within five environments are evaluated, with various clients with varying statistical and demographic distributions, as shown in Figures 2, 3. The scenarios not only allow for a comparison of the performance of the models trained using FL but also allow for the evaluation of the effect that the differences in the underlying statistical and demographic distributions among clients have on the final model. The experiments considered one FL environment with four clients (Scenario 1.2), five heterogeneous FL environments with three clients (Scenario 1.1, Scenario 2, Scenario 3.1, Scenario 3.2, Scenario 4.1), and four heterogeneous FL environments with two clients (Scenario 3.3, Scenario 4.2, Scenario 5.1, Scenario 5.2). Regarding the data distribution across clients, five different cases were investigated: (1) uniform and balanced, where each client had data with the same number of AD and CN patients (Scenario 1.1, Scenario 1.2), (2) skewed and balanced, where clients held data with differing numbers of patients (Scenario 2), (3) uniform and imbalanced in terms of patient's sex, where the clients held data with differing number of patients from particular sex (Scenario 3.1, Scenario 3.2, Scenario 3.3), (4) uniform and imbalanced in terms of patient's age, where the clients held data with differing number of patients from a particular age group (Scenario 4.1, Scenario 4.2), and (5) uniform and non-IID in terms of patient's diagnosis label, where the clients held data with differing number of patients from a particular diagnosis label (Scenario 5.1, Scenario 5.2).

#### 2.3.1 Data and preprocessing

For the experiments, T1-weighted MPRAGE scans from the ADNI database (Petersen et al., 2010), were used. All scans were acquired with 3T scanners at various locations. The scans were pre-processed using the 1mm T1 version of the ICBM152 reference brain as a template after being resampled to a thickness of 1mm. Afterward, Advanced Normalization Tools (ANTs^4^) were used to register the scans to the reference brain template non-linearly. In the end, the FSL Brain Extraction Tool (fsl-bet) (Jenkinson et al., 2012), was used for skull stripping. ADNI is a longitudinal dataset i.e., there are multiple scans of a single subject at several points in time. Thus, the subjects included in the experiments were only subjects that, at the baseline visit, were classified either as Cognitively Normal (CN) patients or patients with AD. The total number of subjects in the experiment was 618 (166 patients with AD, 452 CN patients). There were 345 women and 273 men. The dataset was split into the development (train/validation) and the test set. The split was repeated ten times with ten different random seeds to obtain more robust results. In each training run, the training was repeated five times for all scenarios over the ten splits. The models with the highest performance on the validation dataset were tested on the independent test set. All of the test sets were created once and kept the same until the end of the experiment. The test sets had equal amounts of AD and CN patients (50 AD and 50 CN), with an equal number of women and men of the same diagnosis label and same mean age.

#### 2.3.2 Convolutional neural network architecture

**Figure 4:**
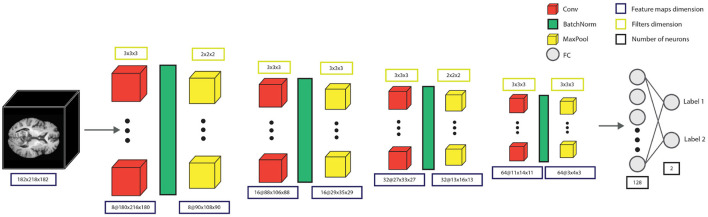
The model consists of four convolutional blocks, each containing a convolutional layer, batch normalization, and max-pooling. After the convolution blocks, there is a fully-connected layer with 128 units, and the output fully-connected layer with two output units, providing the decision for the specific patient.

We investigated the detection of AD using a 3D Convolutional Neural Network (CNN), adapted from Bhle et al. (2019), shown in Figure 4. The model's architecture contained four convolutional blocks, each with one convolutional layer with filter sizes 8, 16, 32, and 64 features, respectively. The convolutional block also had batch normalization and max-pooling with window sizes 2, 3, 2, and 3. The convolutional blocks were followed by two fully connected layers of 128 and 2 units, respectively, where the 2-unit output layer represented the two decision classes, CN and AD. Additionally, before each of the fully connected layers, a dropout of 0.4 was applied. Cross-entropy loss and Adam optimizer were used to train the network with a learning rate and weight decay of 10^−4^.

All federated experiments were performed using DLFi, the in-house built Distributed Learning Framework^5^ at Fraunhofer Heinrich-Hertz-Institute. DLFi is a Privacy-Preserving AI-as-as-Service (PP-AIaaS) solution, providing a training environment for remote clients without transferring the data to a central location. It allows the training of a global model on a set of geo-distributed edge nodes. The federated experiments were demonstrated as a cloud-native application across a multi-server Kubernetes cluster, similar to Shariati et al. (2020).

## 3 Results

**Figure 5:**
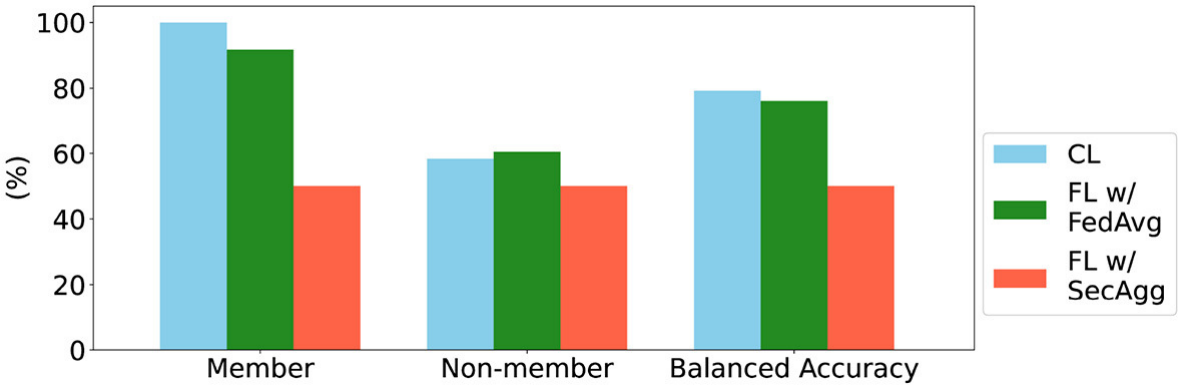
Summary of results for the membership inference attack, based on Shokri et al. (2017). The member and non-member metrics correspond to sensitivity and specificity, and the balanced accuracy is the average of the two.

In Section 3.1, the RPD of the models trained within the ten different learning scenarios using the three learning approaches (CL, FL with FedAvg, and SecAgg) is presented (see Table 1). Section 3.2 gives an overview of the results of the corresponding privacy analysis over the models trained using the three learning approaches (see Figure 5).

**Table 1:** Summary of the obtained average balanced test accuracy (%) with standard deviation for different heterogeneous learning scenarios.

**Scen**.	**Description**	**Num. of clients**	**CL**	**FLw/FedAvg**	**FLw/SecAgg**
1.1	Uniform and balanced	3	83.3 ± 1.6	82.9 ± 1.9	82.1 ± 2.4
1.2	Uniform and balanced	4	83.3 ± 1.6	81.1 ± 2.9	81.0 ± 2.4
2	Skewed and balanced	3	82.6 ± 2.7	81.6 ± 2.2	81.6 ± 2.3
3.1	Uniform and sex imbalanced	3	80.8 ± 2.6	79.0 ± 2.9	79.0 ± 2.7
3.2	Uniform and sex imbalanced	3	80.8 ± 2.6	78.8 ± 2.2	78.1 ± 2.6
3.3	Uniform and sex imbalanced	2	82.2 ± 3.4	81.4 ± 3.5	N/A
4.1	Uniform and age imbalanced	3	78.5 ± 4.1	78.5 ± 3.7	78.1 ± 4.1
4.2	Uniform and age imbalanced	2	74.0 ± 3.2	74.0 ± 3.3	N/A
5.1	Uniform and diagnosis non-IID	3	81.0 ± 2.6	64.2 ± 1.1	N/A
5.2	Uniform and diagnosis non-IID	2	81.0 ± 2.6	78.2 ± 4.4	N/A

### 3.1 Performance comparison

In Table 1, we show the average balanced test accuracy achieved by the models trained using CL, FL with FedAvg, and FL with SecAgg in the ten different learning scenarios. In most cases, the models trained using FL with FedAvg, and with SecAgg achieved balanced test accuracy comparable to those trained centrally. It is important to mention that the environments with two clients were simulated to provide an insight into how the training of the ML model is affected when one of the clients has data from one population, and the other client has data from another population (e.g., one client with only women patients and the other with only men). However, as the SMPC protocol guarantees privacy as long as *N*−1 elements are known (Evans et al., 2018), the smallest number of clients for which SecAgg makes sense is three. So in Scenario 3.3, Scenario 4.2, Scenario 5.1, and Scenario 5.2, we only compare the models trained with FL with FedAvg and CL.

To compare the model performances quantitatively, we use the RPD metric to measure the performance decrease between the federated models and the models trained using CL.

The results show that under the uniform data distribution in Scenario 1.1, the models trained using FL with FedAvg experienced 0.48% RPD, and those trained using FL with SecAgg experienced 1.40% RPD. Scenario 1.1 serves as the simplest, most balanced, pathological example, which shows that in a completely balanced setting, with balanced data distribution in terms of dataset size and diagnosis, the FL approach suffers little performance decrease. Under the extended Scenario 1.2, it can be observed that by adding one more client in the setting (by splitting the same dataset over four instead of three clients), the models trained using FL with FedAvg experienced 2.64% RPD, and the models trained using FL with SecAgg experienced 2.76% RPD. The increase in RPD, in Scenario 1.2 is due to the smaller number of samples per client, as the whole dataset was split over four, instead of three clients as in Scenario 1.1. In Scenario 1.2, we showcase that FL with SecAgg scales well even when we have more than the minimum number of clients for which SecAgg makes sense (three). However, as can be seen, due to dataset size limitation, if the number of clients is too large, the local clients' datasets become very small, making the local objectives very different compared to the global objective, essentially failing the FL process.

Under the skewed balanced data distribution in Scenario 2, both the models trained using FL with FedAvg, and with SecAgg experienced 1.21% RPD. Compared to Scenario 1, Scenario 2 serves as a more realistic example, where different clients have different amounts of data and an imbalanced distribution in terms of diagnosis. However, in this scenario, the models trained using FedAvg and SecAgg also reach accuracy comparable with the centralized approach. This shows that slight variations in the statistical distribution of data among clients, where one of the clients has most of the data, and the other two have a proportion of the rest of the data, only slightly affect the performance of the models trained in the federated setting.

Aside from the difference in the statistical distribution of data among clients, three other demographic variations were explored: sex-based, age-based, and diagnosis-based. The sub-scenarios of Scenario 3, present the effect that imbalanced data distribution in terms of patients' sex has on the models trained in a federated setting. From the experiments, it is observable that the imbalance in terms of patients' sex, does affect the performance of the models trained using FL with FedAvg, and with SecAgg. The effect is the strongest in the sub-scenarios where two clients have the same features (i.e., only women), and one client has a different feature (i.e., only men). Thus, in Scenario 3.1 the RPD is 2.30% for the models trained with both FL with FedAvg, and with SecAgg, and in Scenario 3.2 the RPD is 2.48% for the models trained with FL with FedAvg and 3.34% for the models trained with FL with SecAgg. The model performance decrease is lower in the two-client environment of Scenario 3.3 (RPD of only 1%), where both clients hold a single feature. On the other hand, the sub-scenarios of Scenario 4 present the effect of imbalanced data distribution in terms of patients' age on the models trained in a federated setting. From the experiments, it can be observed that the imbalance in terms of patients' age distribution doesn't affect the performance of the models trained using FedAvg and SecAgg to a great degree. In Scenario 4.1 there is no RPD for the models trained using FL with FedAvg and only 0.50% RPD for the models trained using FL with SecAgg, and in Scenario 4.2 there is no RPD for the models trained using FL with FedAvg. However, as the two age groups had an average age difference of approximately 10 years, it would be interesting to explore whether the same conclusion could be made if the average age difference between the groups was more significant.

Scenarios 5.1 and 5.2 explored the effect of non-IID data on the models trained in a federated setting, as FedAvg has been shown to diverge in practice (McMahan et al., 2017) when data is non-IID. Due to the nature of FL with SecAgg, the fifth scenario only investigated the RPD between the models trained using FL with FedAvg and those trained centrally. In Scenario 5.1, the federated model experienced the highest RPD across all scenarios (RPD of 20.70%). However, the performance of the models trained using FL with FedAvg drastically increased after including a proportion of data from the other diagnosis label in each client's local dataset, as observed in Scenario 5.2 (RPD of only 3.50%). The sub-scenarios of Scenario 5 present the effect that imbalanced data distribution in terms of patients' diagnosis has on the models trained in a federated setting. As the results were very discouraging when having the data split across three clients (i.e., two clients with only CN patients and one client with AD patients), only the environment with two clients is presented. When both clients have completely non-IID in terms of diagnosis, the model trained using FL has the highest performance decrease across all experiments. Scenario 5.2 shows that when data from the second diagnosis label replaces 25% of the data on the client, the relative performance increases by more than 17%.

The RPD values from all of the Scenarios are summarized in Table 2.

**Table 2:** Summary of the RPD (%) between the federated models and the models trained centrally for different heterogeneous learning scenarios.

**Scen**	**RPD for FLw/FedAvg**	**RPD for FLw/SecAgg**
1.1	0.48	1.40
1.2	2.64	2.76
2	1.21	1.21
3.1	2.30	2.30
3.2	2.48	3.34
3.3	1.00	N/A
4.1	0.00	0.50
4.2	0.00	N/A
5.1	20.70	N/A
5.2	3.50	N/A

### 3.2 Privacy analysis

To explore the advantages of FL with SecAgg in terms of privacy and reduction of information loss, we simulated a membership inference attack (Shokri et al., 2017) over the models trained in the uniform and balanced scenario. We present the results of the approach in Figure 5.

**Figure 6:**
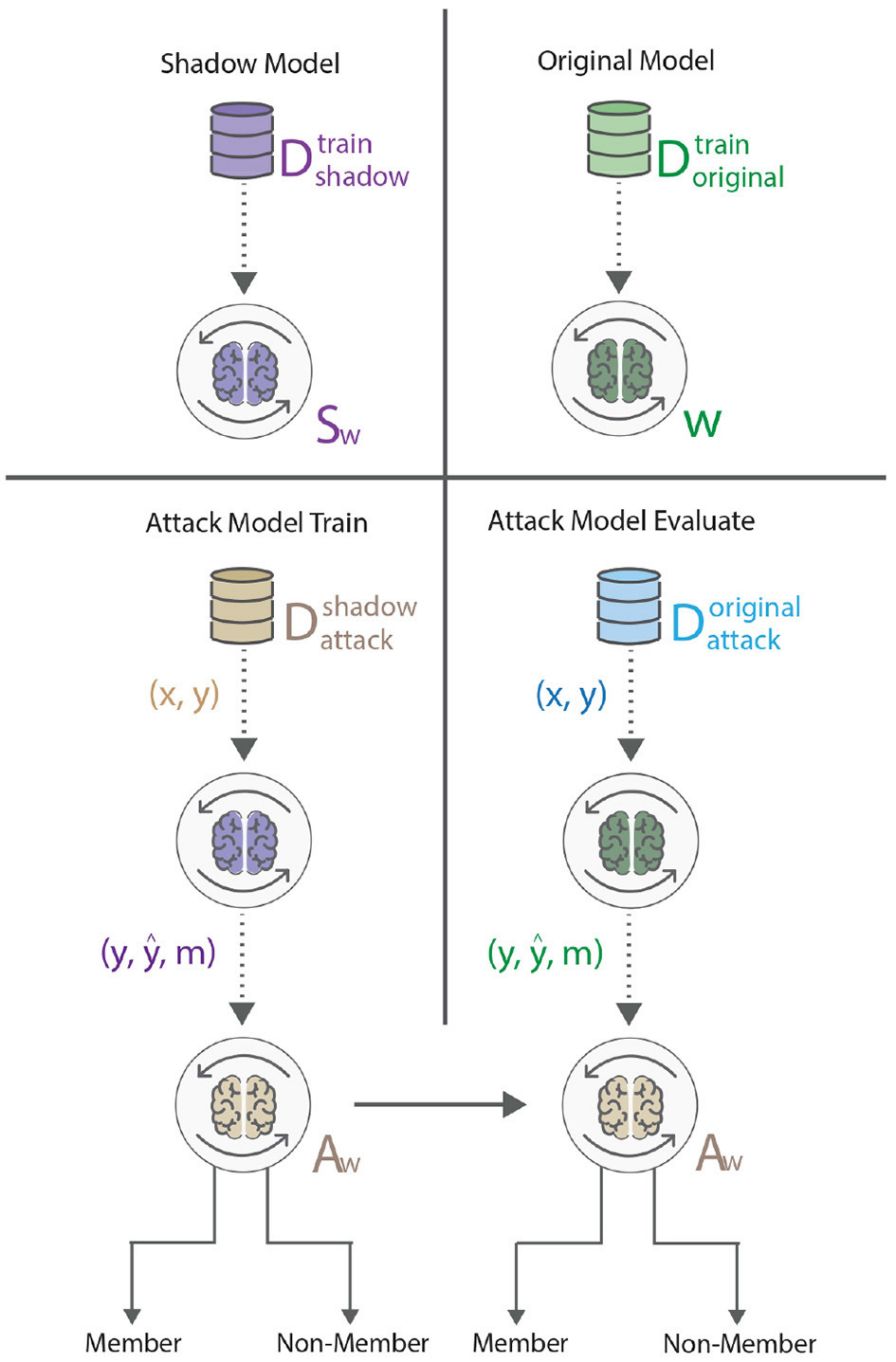
The membership inference attack, based on Shokri et al. (2017), encompasses the training of a shadow model (purple), that behaves the same as the original model (green). Once the shadow model has been trained, the adversary feeds the trained shadow model samples from an auxiliary dataset, and the outputs serve as an input to the attack model (beige). Once the attack model has been trained, it is evaluated on the original model, such that the adversary feeds the original model samples from a second auxiliary dataset and uses the outputs as input to the attack model. In this case, the attack model distinguishes between member and non-member points.

An adversary able to learn whether a particular sample was used to train an ML model indicates information leakage through the model, which possibly leads to a privacy breach (Shokri et al., 2017). Our experiment setup assumes the adversary can access the model architecture and parameters from a particular training epoch. In the federated system, the adversary was assumed to have access to the information available at the central server, or the adversary was the central server itself. The attack is executed by utilizing a shadow model approach as explained by Shokri et al. (2017), where the shadow model is a replica of the original model, trained on a separate dataset $D_{shadow}^{train}$, disjoint from the training dataset of the original model $D_{original}^{train}$. The idea behind the setup can be observed in Figure 6. Such a setup presents a more realistic environment, as one cannot assume that the attacker has access to some samples from the training dataset of the original model.

From the results it is observable that the attack was successful for the models trained using CL and the models trained in a federated setting with FedAvg. For the model trained with SecAgg, the attack model was unsuccessful in determining which data samples were part of the training set of a particular client. This result is consistent with the privacy guarantee of SecAgg when trying to infer information from the clients' local model updates, from the perspective of the central server (Evans et al., 2018). In general, this is the expected behavior, and a range of studies have looked into the information leakage of models trained using FL, reaching the same conclusion (Nasr et al., 2019; Zari et al., 2021).

## 4 Discussion

In this work, we introduced a secure FL architecture for training ML models for AD detection based on sMRI data. We empirically demonstrated the effectiveness, as well as the convergence and privacy guarantees of the models trained using FL with FedAvg, and with SecAgg in different heterogenous environments with varying demographical and imbalanced data distributions. Through the simulation of ten scenarios within five environments, we showed that the model performance obtained when using FL with FedAvg and with SecAgg is on par with the models trained centrally; however certain demographic and imbalanced data distributions affected the performance of the models to a greater degree. First, we showcased that slight variations in the statistical distribution of data among clients, such as in Scenario 2, where one of the clients has most of the data, and the other two have a proportion of the rest of the data, only affected the performance of the models trained in the federated setting to a small degree. Regarding the models trained in environments where the data is uniform but imbalanced in terms of sex and age, we observed that the patient's sex imbalance affected the federated models to a greater degree than the imbalance in terms of the patient's age. Last, through the sub-scenarios of Scenario 5, we observed that the models trained in an environment where the clients held data with differing number of patients from a particular diagnosis label, suffered the greatest RPD across all the experiments. This showed that when the data is non-IID in terms of diagnosis labels across the clients, the effect on the FedAvg algorithm is the largest.

The model performance obtained when using FL with SecAgg across the experiments is on par or the same as the model performance of the models trained without SecAgg. However, based on individual data splits, the models trained with the addition of SecAgg show higher variability. This is the expected behavior because some information loss occurs when the model parameter updates are encoded to fixed-point precision and correspondingly decoded to float-point precision. Despite this disadvantage, FL with SecAgg offers additional advantages in terms of privacy and reduces the risk of information loss. To explore these privacy guarantees, we also simulated a membership inference attack (Shokri et al., 2017) over the models trained in the uniform and balanced scenario. The models trained with FL with SecAgg, were shown to protect the data on the level of the client from the perspective of the central server, where the adversary is not able to learn any particular information regarding the training data. If the adversary would not target a specific client in the federated setting, both with and without Secure Aggregation, the accuracy of the attack would be theoretically the same as the accuracy for targeting the model trained centrally. However, in the experiments, only a single client is targeted to explore how much information leakage is associated with a single client's local model update.

Overall, the experiments showed that FL with SecAgg is a practical approach that can be used when training high-quality models on the task of AD detection. While FL offers many advantages, the addition of SecAgg allows for developing a client-based privacy-preserving approach.

### 4.1 Related work

Several studies have demonstrated the proof-of-concept application of FL to real-world neuroimaging data. Mainly, FL has been used for whole-brain segmentation in MRI (Roy et al., 2019; Rieke et al., 2020), brain tumor segmentation (Sheller et al., 2018; Li et al., 2019; Rieke et al., 2020), brain-age prediction (Stripelis et al., 2021a,b), etc. Silva et al. (2019), presented a method for utilizing multi-site neuroimaging data to analyze different neurological diseases. They studied brain structural relationships across diseases and clinical cohorts using synthetic data and then applied the framework to multi-database studies, such as ADNI.

Recently, Stripelis et al. (2022) have also presented an FL architecture that utilizes Homomorphic Encryption (HE) to train ML models for AD detection. The authors studied several prominent AD datasets, splitting the dataset heterogeneously over clients only in terms of dataset sizes. However, HE protocols relying on key-based cryptography have been shown to usually experience high computational complexity for implementing neural network training and inference (Kaissis et al., 2021).

### 4.2 Limitations

One of the most crucial challenges of any FL system is the privacy provided to the clients. Our system assumes an honest but curious setting and doesn't provide any additional protection against active adversaries that could participate directly in training and poison the model that is being trained. Although SecAgg provides comprehensive privacy protection on the client level, it doesn't guarantee to the clients that the shared model is the one promised by the central server (Kaissis et al., 2021). Additionally, SecAgg doesn't fully protect against information leakage on the level of the global model, only on the level of local models from a particular client. Differential Privacy (DP) could be used for additional protection of the data, by adding noise to the results at a particular stage of model training. DP has been used, both on models trained centrally (Abadi et al., 2016), and with FL (Choudhury et al., 2019). DP offers three locations of application in different stages of the model training: adding noise to the input, adding noise to model parameters, and perturbing the objective function (Choudhury et al., 2019; Zhao J. et al., 2019). DP has been shown to provide defense against membership inference attacks, by perturbing the model parameter weights (Chen et al., 2020). Extending the current work with DP would protect on the level of the global model. While DP adds a level of privacy, it could destroy useful information in different stages of the training, which is a common research question. DP with FL has been used in many studies, all showing promising results, and some have even implemented the combination of SMPC and DP for additional data protection (Kaissis et al., 2021). Thus, extending the current work with DP is an exciting task for future work.

As could be observed from the sub-scenarios of Scenario 5, the non-IID data in terms of diagnosis across clients affects FL strongly. Thus future work should also address the challenge of non-IID data across clients. Several studies have provided concepts for novel federated aggregation algorithms that try to cope with the effect of non-IID data. For example, Li et al. (2020b), proposed FedProx to deal with the heterogeneity in federated settings. FedProx is a generalization and re-parametrization of the FedAvg algorithm that allows for a variable amount of work to be performed locally across clients, depending on the available system resources. The authors provided convergence guarantees using bounded dissimilarity assumption and demonstrated that in highly heterogeneous settings, they can improve the testing accuracy by 22% on average. Yu et al. (2020), presented another class of methods for robust, personalized FL. Their aggregation framework can handle non-IID data, outliers, and clients that transmit their local updates late. It doesn't require all of the clients to agree on a single standard model. The authors provided a convergence analysis and concluded that their method converges for convex, non-convex problems, robust aggregation, and the case when clients transmit their local model updates late. These approaches are all promising solutions for non-IID data's challenges to the currently used aggregation mechanism.

Additionally, even though compared to the literature, the results that are presented are in line with the baseline results [76%–91% for discriminating AD and CN patients (Wen et al., 2020)]; however, they could have been improved by exploring more methods for data augmentation and hyper-parameter tuning. Since more than 1,000 runs of the experiment were made to obtain robust results, exploring all of these in detail was not possible from a computational point of view.

## 5 Conclusion

In conclusion, in this work, we empirically demonstrated the effectiveness of using FL with FedAvg, and with SecAgg to train ML models for AD detection based on sMRI data. The results obtained via the simulated heterogeneous environments with varying demographical and imbalanced data distributions highlighted the importance of studying such statistical differences when training an ML model for AD detection using FL. Additionally, through simulated membership inference attacks, we showcased the privacy guarantees of SecAgg, proving the additional advantages of SecAgg in terms of privacy and reduced risk of information loss.

Our immediate future work includes expanding the FL architecture with DP to provide a privacy guarantee on the level of the global model. We also plan to explore more efficient aggregation methods to mitigate the effect of non-IID data on the models trained in the federated setting, as seen in Scenario 5.1. Moreover, when more public data is available, we could explore how a male patient-dominated environment in Scenario 3 and a larger mean age difference in Scenario 4 would affect the results.

## Data availability statement

The original contributions presented in the study are included in the article/Supplementary material, further inquiries can be directed to the corresponding author.

## Author contributions

AM: Conceptualization, Investigation, Methodology, Software, Writing—review & editing, Writing—original draft. PS: Conceptualization, Investigation, Methodology, Software, Writing—review & editing, Supervision. KR: Conceptualization, Investigation, Methodology, Writing—review & editing, Supervision. BS: Conceptualization, Investigation, Methodology, Writing—review & editing, Supervision, Funding acquisition, Project administration. JF: Conceptualization, Investigation, Methodology, Writing—review & editing, Supervision, Funding acquisition, Project administration.

## Footnotes

^1^ https://www.hhs.gov/hipaa/for-professionals/security/laws-regulations/index.html

^2^ https://gdpr-info.eu/

^3^ adni.loni.usc.edu

^4^ http://stnava.github.io/ANTs/

^5^www.hhi.fraunhofer.de/dlfi.
