# Supplementary material for: Secure federated learning for Alzheimer's disease detection
Source: Front Aging Neurosci. 2024 Mar 7;16:1324032. doi: 10.3389/fnagi.2024.1324032 (PMC10954782; doi:10.3389/fnagi.2024.1324032)
Supplement: Supplementary file 1 [file Data_Sheet_1.pdf]

Supplementary Material

1 SUPPLEMENTARY TABLES AND FIGURES

1.1 Figures

Algorithm 1 Federated Averaging algorithm. There are N clients, indexed with i. B represents the set of data batches on a client, E is the number of training epochs on a client, and η is the learning rate.

Central Server:

  initialize ω_0

  for each round $r = 1, 2, ...$ **do**

    for each client P_i **in parallel do**

      $\omega^i_{r+1} \leftarrow ClientUpdate(i, \omega_r)$

    $\omega_{r+1} \leftarrow \sum_{i=1}^{N} [(1/N) * \omega^i_{r+1}]$

ClientUpdate(i, ω) :

  for each local epoch e from 1 to E do

    for batch $b \in B$ **do**

      $\omega \leftarrow \omega - \eta \nabla l(\omega; b)$

return ω **to server**

Figure S1. A typical implementation of the FedAvg algorithm. From the algorithm, it can be observed that each client computes the local gradient update on their local data, and the central server performs the gradient aggregation and the update of the global model.

Algorithm 2 Secure Aggregation utilising SMPC. There is a list of $\chi = \{1, 2, ...N\}$ clients, indexed with i. *mod* is the modulo operation. rand.range means random numbers in a specific range. l is the loss for the training and $\mathcal{L}$ is the average loss over a batch. B represents the set of data batches on a client, E is the number of training epochs on a client, R is the number of rounds and η is the learning rate. y_n is the ground truth label, and $\hat{y_n}$ is the predicted label.

send initial model ω_0 to all clients
    for each **round** r in R:
        on each client i **in parallel do**:
            for each **epoch** e in E:
                for batch $b \in B$:
                    compute the model output $\hat{y_n}$
                    $\mathcal{L}(\omega_i^r) = \frac{1}{|b|}\sum_{n=1}^{|b|} l(\omega_i^r; \hat{y_n}; y_n)$
                    $g_i = \nabla_{\omega_i}\mathcal{L}(\omega_i^r)$
                    $\omega_i^r \leftarrow \omega_i^r - \eta g_i$
            to secret-share ω_i^r among all clients **do**:
                encode ω_i^r values to fixed precision
                consider Q as a very large prime number
                for $j \in \{1, ..., N-1\}$ **do**:
                    from rand.range(0, Q) create $\omega_i^{r,j}$
                    $\omega_i^{r,N} = mod(\omega_i^r - \sum_{j=1}^{N-1} \omega_i^{r,j}, Q)$
                for j in χ **do**:
                    send the shares $\omega_i^{r,j}$ to the client j
        when each client j receives all shares **do**:
            $\omega_j^{r,T} = \sum_{i=1}^{N} \omega_i^{r,j}$
            send $\omega_j^{r,T}$ to the central server
        when the central server receives all collective shares **do**:
            decode all $\omega_j^{r,T}$ to float precision
            $\omega_g^r = \frac{1}{M}\sum_{i=1}^{N}\omega_i^{r,T}$
            send ω_g^r back to each client

Figure S2. An implementation of the FL with SecAgg algorithm. The algorithm presents a mathematical overview of how SPDZ is utilized in an FL scheme using SecAgg.
